# Cognitive Development in Children with Autism Spectrum Disorder and the Moderating Role of Intervention and ASD Persistence

**DOI:** 10.3390/bs15111445

**Published:** 2025-10-23

**Authors:** Maya J. Golden, Lianna R. Lipton, Georgios Sideridis, Stephanie J. Brewster, William Barbaresi, Elizabeth Harstad

**Affiliations:** 1 Division of Developmental Medicine, Boston Children’s Hospital, Boston, MA 02115, USA; maya.golden@childrens.harvard.edu (M.J.G.); georgios.sideridis@childrens.harvard.edu (G.S.); stephanie.brewster@childrens.harvard.edu (S.J.B.); william.barbaresi@childrens.harvard.edu (W.B.); 2 Rosamund Stone Zander and Hansjoerg Wyss Translational Neuroscience Center, Boston Children’s Hospital, Boston, MA 02445, USA

**Keywords:** autism spectrum disorder/ASD, cognitive outcomes, intervention history, Bayley Scales of Infant and Toddler Development

## Abstract

This study examined whether Bayley Scales of Infant and Toddler Development-III (Bayley-III) standardized cognitive scores from toddlers diagnosed with autism spectrum disorder (ASD) predict intellectual quotient (IQ) at early school age and whether ASD persistence or interventions received moderate this relationship. Children diagnosed clinically with ASD at 12–36 months underwent research assessments at 5–7 years. Of 212 children diagnosed as toddlers, 133 continued to meet DSM-5 ASD criteria based on current functioning at school age (“persistent ASD”), and 79 did not (“non-persistent ASD”). A moderate positive correlation was found between baseline cognitive scores in toddlerhood and school age IQ (r (210) = 0.45, *p* < 0.001). Children with baseline cognitive scores < 70 showed greater variation in school age IQ compared to those with baseline scores > 85. Non-persistent ASD status was associated with a higher rate of cognitive change from toddler to school age (S_diff_ = 15.044; z = 4.432, *p* < 0.001). Overall, 94.3% of the sample received ASD-specific interventions. There was no relation between hours of ASD-specific interventions and change in cognitive trajectories for children with non-persistent ASD and an inverse relationship for children with persistent ASD.

## 1. Introduction

Children who are diagnosed with autism spectrum disorder (ASD) before 36 months of age frequently present with early developmental delays (Christensen et al., 2018). Both ASD symptoms and developmental levels of children diagnosed with ASD at a young age may change over time (Elias & Lord, 2022; Joseph et al., 2002). Understanding the extent to which developmental levels may change over time for a young child with ASD, and the potential moderating role of ASD characteristics, may help to inform clinicians and parents about prognosis at the time of initial ASD diagnosis.

The Bayley Scales of Infant and Toddler Development, Third Edition (Bayley-III; Albers & Grieve, 2007) is an assessment tool used to characterize developmental status in children aged 1–42 months. Previous studies have examined the association between Bayley cognitive standard scores at a young age and intellectual quotients (IQs) several years later. Research on the use of the Bayley-III in children born preterm or with very low birth weights have shown mild to moderate correlations between early cognitive scores and later IQ (Flynn et al., 2020; Lowe et al., 2023; Månsson et al., 2021; Nishijima et al., 2022). However, these studies also demonstrated that the Bayley-III can fail to predict later diagnoses of intellectual disability or misclassify children who do not have intellectual disability. An older study of children with ASD found a moderate positive correlation between developmental quotient scores from the Bayley Infant Scales of Development at a mean age of 3 years and 2 months and IQ scores at a mean age of 6 years and 4 months (Lord & Schopler, 1989). Cognitive trajectories in children diagnosed with ASD vary; one publication reported that 75% of the participants experienced some relative improvement in IQ from early to middle childhood, while about 25%, who started out with significantly lower cognitive levels, had a decrease in IQ (Solomon et al., 2017). Additional research is needed to better understand the relationship between initial developmental level at the time of ASD diagnosis in toddlerhood and IQ at early school age.

ASD symptoms can decrease over time, with some children no longer manifesting significant ASD symptoms several years after initial ASD diagnosis (Fountain et al., 2023; Georgiades et al., 2022; Giserman-Kiss & Carter, 2020; Moulton et al., 2016). Our group previously reported that up to 37% of children diagnosed clinically with ASD at 12–36 months of age did not meet criteria for ASD based on their current functioning when re-evaluated for a research assessment at school age (termed “non-persistent ASD”) (Harstad et al., 2023). Some studies have found no association between baseline cognitive functioning and ASD persistence, while others have found that children with non-persistent ASD had significantly higher cognitive levels at baseline compared to children with persistent ASD (Barbaro & Dissanayake, 2017; Chawarska et al., 2009; Hinnebusch et al., 2017; Lord et al., 2006; Moulton et al., 2016; Turner & Stone, 2007; Zachor & Ben-Itzchak, 2020). Thus, it remains unclear whether baseline cognitive functioning is truly predictive of ASD persistence, and it is not known whether cognitive trajectories differ significantly for children with persistent vs. non-persistent ASD.

The impact of interventions received after the initial ASD diagnosis on the trajectory of cognitive development is also unclear. For children with an ASD diagnosis under 3 years of age, ASD-specific therapies, such as Applied Behavioral Analysis (ABA) and the Early Start Denver Model (ESDM), are often recommended (Dawson et al., 2010; Shire et al., 2017; Zwaigenbaum et al., 2015). These interventions are generally recommended to occur at high levels of intensity (>20 h/week) (Applied Behavior Analysis (ABA)|Autism Speaks, n.d.; Dawson et al., 2010). One study from 2014 reported that significantly more children with non-persistent ASD had received ABA therapy before 3 years of age than children with persistent ASD, although the intensity of ABA did not vary between groups (Orinstein et al., 2014). Since that time, the overall receipt of ABA and other ASD-specific interventions has risen, due in part to increased insurance coverage (Grosse et al., 2020). Thus, it is unclear whether there are discrepancies in receipt of interventions for children with non-persistent and persistent ASD in a contemporary sample. There have been variable findings related to the impact of receipt (yes/no) and intensity (hours) of ASD-specific interventions on cognitive functioning. Some studies suggest that receipt of ABA and the ESDM in early childhood leads to notable gains in IQ, with a higher intensity of services leading to greater gains in IQ (M. Clark et al., 2023; Gitimoghaddam et al., 2022; Godel et al., 2022; Lovaas, 1987; McEachin et al., 1993; Shi et al., 2021). Other studies have not demonstrated these impacts of ASD-specific interventions on IQ (Sandbank et al., 2024; Yu et al., 2020).

This study aims to determine whether Bayley-III cognitive scores at the time of clinical ASD diagnosis at 12–36 months old predict later standardized IQ scores at 5–7 years of age. Additionally, we examined whether this relationship is moderated by persistence of ASD or hours of ASD-specific interventions received in the 36 months following ASD diagnosis.

## 2. Materials and Methods

### 2.1. Recruitment and Study Subjects

The present study is based on a previously described cohort of 213 children who were diagnosed with ASD as toddlers and re-evaluated with a research assessment at school age (Harstad et al., 2023). This study recruited children ages 5 years and 0 months to 7 years and 11 months old who previously received a DSM-5 ASD diagnosis between ages 12 and 36 months by a multi-disciplinary clinical team at a single outpatient developmental–behavioral pediatric clinic. Children who were non-English-speaking, in custodial care, and/or had genetic conditions known to affect neurodevelopmental outcomes (e.g., Down syndrome, Fragile X syndrome, or PTEN mutation) were excluded. Eligible participants were recruited for a follow-up research assessment at school age via mail, phone calls, and in-person recruitment. This study received ethical approval from the Boston Children’s Hospital IRB. Caregivers gave informed written consent. Of the 213 children in the primary study, 1 did not have standardized cognitive scores reported at the time of clinical ASD assessment, and thus 212 children were included in the current analyses.

### 2.2. Clinical Multi-Disciplinary ASD Diagnosis at 12–36 Months Old (Time 1)

Each child was seen by a board-certified developmental–behavioral pediatrician (DBP) and a doctoral level child psychologist for a clinical ASD diagnostic assessment at age 12–36 months (Time 1). All initial clinical assessments took place in person between July 2013 and July 2018, more than one year before the COVID-19 pandemic. During the Time 1 clinical ASD assessments, psychologists administered the Bayley-III and Autism Diagnostic Observation Schedule, Second Edition (ADOS-2; Lord et al., 2012). Immediately following each clinical assessment, the DBP and psychologist had consensus meetings, and all children received a clinical DSM-5 ASD diagnosis at Time 1. For study analyses, we used the cognitive standard scores from the Bayley-III that were abstracted from the medical records, hereafter referred to as the cognitive score at Time 1.

### 2.3. Research Assessment at Age 5–7 Years Old (Time 2)

Research assessments were conducted between August 2018 and January 2022 (Time 2). They included the Autism Diagnostic Interview-Research (ADI-R; Lord et al., 1994), ADOS-2 (Module 1, 2, or 3), and Differential Abilities Scales-Second Edition (DAS-II; Elliott et al., 2018) or Bayley-III for the 16 children unable to complete the DAS. For analyses, DAS-II Full Scale IQ Scores and Bayley Cognitive scores were used to determine the child’s IQ at Time 2, with Bayley Cognitive age equivalents being translated into developmental quotients to approximate IQ level. All assessments were conducted by research psychologists or supervised research assistants trained and research-reliable in administration of the ADOS-2 and ADI-R. Following the completion of each research assessment, an experienced research psychologist gave participants an overall best-estimate research diagnosis of DSM-5 ASD using all available information from diagnostic testing and behavioral observations (dichotomized to persistent ASD or non-persistent ASD based on current functioning). Because the COVID-19 pandemic began during this study, 111 of the 213 research assessments were completed with facemasks. ADOS-2 assessment procedures with facemasks were performed as close to standardized ADOS-2 administration as possible. Use of facemasks had minimal to no impact on the overall assessment results in this sample (Peisch et al., 2024).

### 2.4. Demographics and Interventions

Parents provided demographic information including the child’s race/ethnicity, family income, and maternal education level through questionnaires completed at the research assessment. Parents also reported type and hours per week of interventions received at 6-month intervals from age 1.5 years until the time of research assessment. Interventions recorded at each interval included ABA (Cooper et al., 2020), the ESDM (Dawson et al., 2010), Floortime (Hess, 2020), Social Communication/Emotional Regulation/Transactional Support (SCERTS; Prizant et al., 2006), and Relationship Developmental Intervention (RDI; Gutstein et al., 2007). For this study, interventions were analyzed in two categories: (1) ABA and (2) ASD-specific interventions (ABA, ESDM, Floortime, SCERTS, RDI).

### 2.5. Data Analyses

We used frequencies and descriptive statistics to describe the sample. To examine the relationship between Time 1 cognitive score and Time 2 IQ across the entire sample of 212 children, we employed a Pearson correlation. The mean Time 1 cognitive scores and mean Time 2 IQ scores for children with persistent and non-persistent ASD were used to calculate changes in cognition from Time 1 to Time 2 for the two groups. Intercept and slope difference z-tests in Mplus 8.9 were used to assess the differences in initial cognitive scores and change in cognition from Time 1 to Time 2 between children with persistent and non-persistent ASD (Muthén & Muthén, n.d., 1998–2017).

For ABA and ASD-specific interventions, we calculated the percentages of children with persistent ASD and non-persistent ASD receiving interventions at 11 different 6-month intervals post ASD diagnosis (0–6, 6–12, 12–18, 18–24, 24–30, 30–36, 36–42, 42–48, 48–54, 54–60, and 60–66 months). Chi-square tests of independence were used to determine whether there were relationships between receipt of interventions (ABA and ASD-specific; yes/no) and ASD persistence at each time interval.

A two-level random-slopes regression model in Mplus 8.9 was used to examine the moderating role of hours of intervention (ABA and ASD-specific) in change in cognition across children with persistent ASD and non-persistent ASD (Muthén & Muthén, n.d., 1998–2017). For the within-person (Level-1) model, the intra-individual trajectory of cognitive scores was modeled as a function of time. Random intercepts and slopes from the within-person model were then used as outcome variables at the between-level (Level-2) model to test for the 2-way interaction as a function of ASD persistence and a 3-way interaction as a function of ABA exposure. The model was applied separately to 6 different 6-month intervals of interventions received post ASD diagnosis (0–6, 6–12, 12–18, 18–24, 24–30, and 30–36 months). We initially attempted to evaluate interventions received from Time 1 to Time 2 (up to 66 months post ASD diagnosis); however, the model could not be run due to small amounts of variability in interventions at later time intervals. Thus, given the emphasis placed on receiving interventions soon after an ASD diagnosis for young children, and to minimize the confounding relationship between children naturally receiving fewer interventions as they make positive progress, we focused on interventions received up to 36 months after ASD diagnosis. For each interval, cognitive scores and intervention exposure were aggregated within the intervention time intervals, allowing for time-specific growth estimation and moderation analysis across groups and hours of ABA. The model incorporated full information maximum likelihood estimation with robust standard errors using the Mplus MLR estimator, and missing data were handled via pairwise deletion for the specified analysis variables.

Power for the two-level random-slopes regression model was estimated using a Monte Carlo simulation with the effect of interest being the difference in slopes between non-persistent and persistent groups as a function of one unit of change in interventions. Using a level of significance equal to 5% for a two-tailed test and 1000 replicated samples, the power of the slope estimate of 0.5 standard deviation was 99.9%. Thus, ample levels of power were present to estimate the random slope.

## 3. Results

Among the 212 children (17% female; 83% male) diagnosed clinically with DSM-5 ASD at 12–36 months (Time 1), the mean age at the time of the research assessment was 74.3 months (SD = 7.1; Time 2). The majority of children were White (80%), had private insurance (70%), and had a maternal report of a bachelor’s degree or higher level of education (68%; Table 1). Seventy-two percent of the 190 children who provided income data reported an annual household income of USD >81,000, which is the median income in Massachusetts (United States Census Bureau, 2020).

### 3.1. Relationship Between Time 1 (Toddler) Cognitive Scores and Time 2 (School Age) IQ

**Figure 1:**
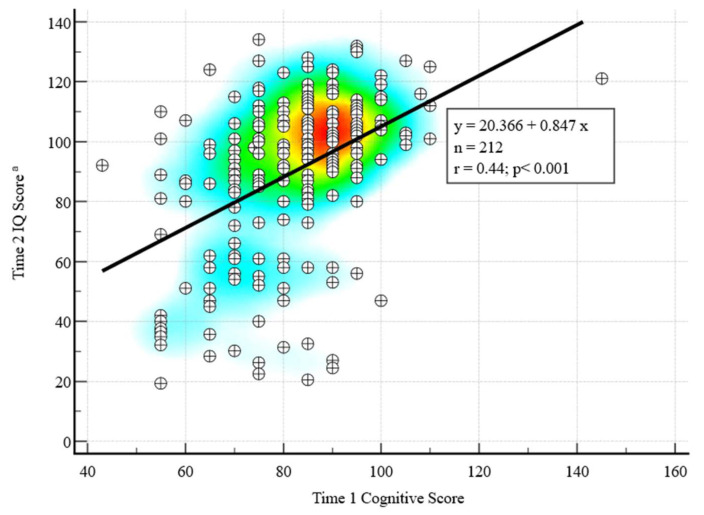
Correlation between Time 1 (toddler) and Time 2 (school age) cognitive scores with heat map. Warmer colors indicate higher correlations between Time 1 and Time 2 scores. IQ = Intellectual quotient. ^a^ N = 196 completed Differential Abilities Scales, Second Edition (DAS-II) at Time 2; for the N = 16 who completed the Bayley Scales of Infant and Toddler Development, Third Edition (Bayley-III) at Time 2, a developmental quotient was calculated as a proxy for Time 2 IQ score.

The Time 1 mean Bayley Cognitive standard score was 81.7 (SD = 14.0); 16.0% scored < 70 and 4.2% scored > 100 (Table 2). The Time 2 mean IQ was 89.8 (SD = 26.4); 21.7% scored < 70 and 43.4% scored > 100 (Table 2). A significant positive correlation was found between Time 1 cognitive scores and Time 2 IQ, r (210) = 0.45, *p* < 0.001 (Figure 1). Of children with Time 1 cognitive scores < 70 (N = 34), 55.9% had Time 2 IQ < 70, and 11.8% had IQ > 100, with the rest in between these scores. Of children with Time 1 cognitive scores 86–100, the majority (83.2%) had Time 2 IQ > 85. Of children with Time 1 cognitive scores >100 (N = 9), all had Time 2 IQ > 85, and 8 had IQ >100.

**Figure 2:**
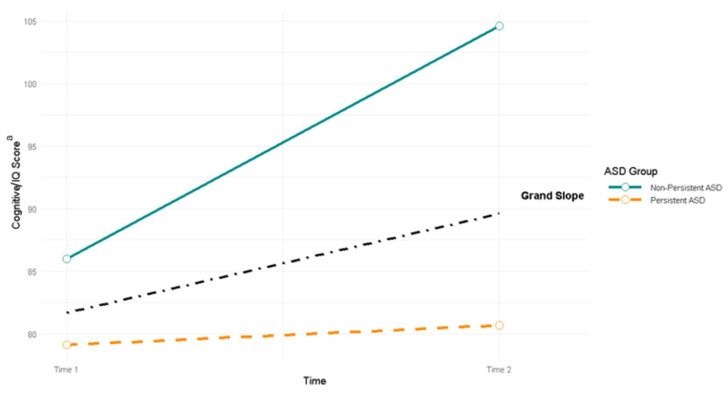
Change in cognitive/IQ scores from Time 1 (toddler) to Time 2 (school age) for children with persistent vs. non-persistent ASD (N = 212, N_Non-Persistent_ = 79, N_Persistent_ = 133). ^a^ Cognition at Time 1 was determined by cognitive standard scores from Bayley-III. Cognition at Time 2 was determined by the child’s approximate IQ level based on DAS-II full scale IQ scores or Bayley-III cognitive scores translated into developmental quotients (for the N = 16 who completed Bayley-III at Time 2).

Time 1 cognitive scores were significantly higher for children with non-persistent ASD (86.0, SE = 1.3; N = 79) than for children with persistent ASD (79.1, SE = 1.3; N = 133), z = 3.80, *p* < 0.001. Importantly, the mean slope of change of Time 1 cognitive score to Time 2 IQ score was significantly steeper for children with non-persistent ASD (18.6, SE = 1.8) than for children with persistent ASD (1.6, SE = 2.2), Sdiff = 17.0; z = 5.91, *p* < 0.001 (Figure 2).

### 3.2. Receipt of ABA and Any ASD-Specific Interventions

**Figure 3:**
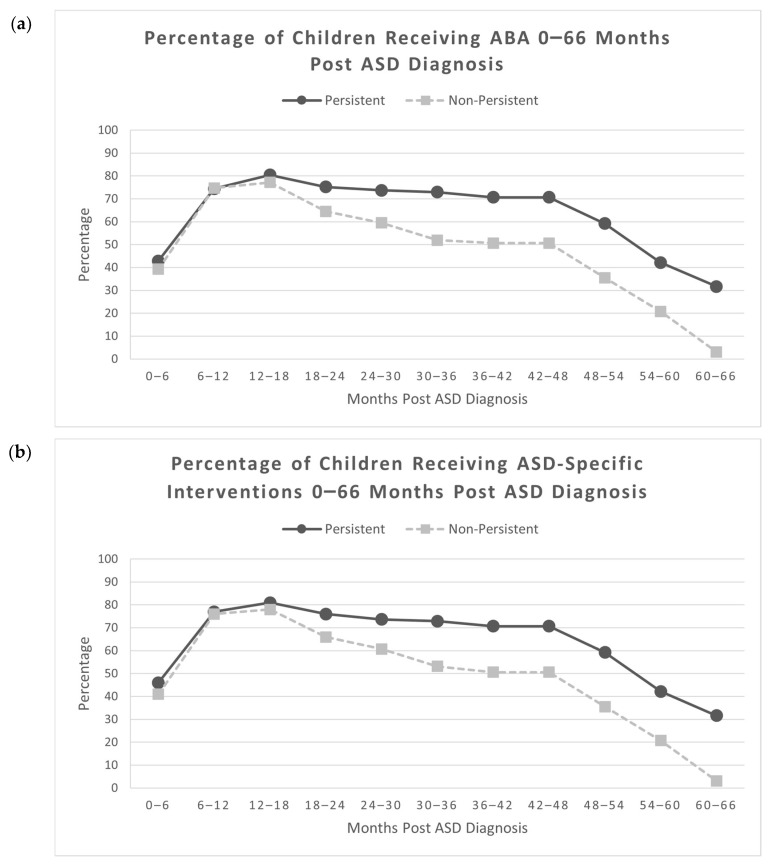
(**a**) Percentage of children receiving ABA 0–66 months post ASD diagnosis. ABA = Applied Behavioral Analysis; (**b**) percentage of children receiving ASD-specific interventions 0–66 months post ASD diagnosis (N = 212, N_Non-Persistent_ = 79, N_Persistent_ = 133).

The percentage of children receiving ABA in both the persistent and non-persistent groups increased continuously from 0 to 18 months post ASD diagnosis and then decreased continuously from 18 to 66 months post ASD diagnosis (Figure 3a). ABA was overwhelmingly the most frequently received type of ASD-specific intervention (Berg et al., 2024); of the 212 children in this study, 192 received only ABA in the intervals from 0 to 36 months after initial ASD diagnosis, 8 received ABA plus other ASD-specific interventions (ESDM, Floortime, SCERTS, RDI), and 5 received only other ASD-specific interventions (no ABA; ESDM, Floortime, SCERTS, RDI). Thus, the same trend was observed for the overall rate of any ASD-specific interventions (Figure 3b).

There was no difference between the percent of children with persistent ASD (95.5%) and non-persistent ASD (88.6%) who received ABA during at least one time period from 0 to 66 months post ASD diagnosis, although the difference approached significance, χ^2^ (1, N = 212) = 3.57, *p* = 0.059. Significantly more children with persistent ASD (97.0%) received any ASD-specific interventions during at least one time period between 0 and 66 months post ASD diagnosis compared to children with non-persistent ASD (89.9%), χ^2^ (1, N = 212) = 4.70, *p* = 0.030, Cramer’s V = 0.149, a small effect size. There were no significant differences in ABA receipt by children with persistent ASD and children with non-persistent ASD from 0 to 24 months post ASD diagnosis (Table 3). Between 24 and 66 months post ASD diagnosis, significantly more children with persistent ASD were receiving ABA in comparison to children with non-persistent ASD, with small effect sizes. From 54 to 66 months post ASD diagnosis, fewer than half of the children with persistent ASD (32–42%) and fewer than a quarter of the children with non-persistent ASD (3–21%) were receiving any ASD-specific interventions (Table S1).

### 3.3. Change in Cognitive Scores as a Function of Hours of ABA and ASD-Specific Interventions Received

A two-level random-slopes model was estimated to examine individual differences from Time 1 cognitive score to Time 2 IQ for children with non-persistent ASD (N = 79) and children with persistent ASD (N = 133) as a function of hours of ABA received at different 6-month intervals following clinical ASD diagnosis. From 0 to 6 and 6 to 12 months following clinical ASD diagnosis, there were no significant effects of hours of ABA received on the slopes of cognitive change for children with persistent and non-persistent ASD (see Table 4). At all 6-month time intervals from 12 to 36 months post ASD diagnosis, receipt of ABA by children with persistent ASD was associated with a less positive trajectory of cognitive scores compared to children with persistent ASD who did not receive any ABA. However, among children with non-persistent ASD, there was no significant effect of receipt of ABA at any of the time intervals. When applying the same model to hours of receipt of any ASD-specific interventions (i.e., not limited to ABA), the results replicated those for receipt of ABA (see Table 5).

## 4. Discussion

The present study examined the relationship between Bayley-III cognitive standard scores from clinical ASD evaluations at ages 12–36 months and standardized IQ scores from research assessments at 5–7 years old for 212 children initially clinically diagnosed with ASD. Additionally, this study investigated whether ASD persistence and hours of ABA/ASD-specific interventions moderated change in cognitive trajectories. There was a moderately positive correlation between Time 1 Bayley cognitive scores and Time 2 IQ scores, such that in the overall sample, improvements in measures of cognition were found over time. When examining cognitive development separately for children with persistent versus non-persistent ASD, ASD persistence was significantly correlated with this change in trajectory; children with non-persistent ASD had significantly higher Time 1 cognitive standard scores and showed greater gains in cognitive trajectories from Time 1 to Time 2 compared to children with persistent ASD. Children with persistent and non-persistent ASD received ABA and ASD-specific interventions at similar rates after initial clinical ASD diagnosis up to 24 months after diagnosis, after which children with non-persistent ASD were less likely to receive interventions than those with persistent ASD. Across the 6-month timepoints in the 36 months after initial clinical ASD diagnosis, there was no effect of hours of ABA/ASD-specific interventions received at each timepoint on the change in cognitive trajectories for children with non-persistent ASD. However, for children with persistent ASD, there was an inverse relationship between the rate of positive cognitive change and number of hours per week of ABA/ASD-specific interventions received between 12 and 36 months after clinical ASD diagnosis.

Consistent with past research on cognitive development in children with ASD, we found that for most children, cognitive skills tend to increase over time (Solomon et al., 2017; Yang et al., 2011). To our knowledge, this study is one of the first to look at the ability of the Bayley-III to predict cognitive trajectories at early school age in a large cohort of children initially diagnosed with ASD in toddlerhood, and the results are similar to studies on children born preterm or with very low birth weights (Flynn et al., 2020; Lowe et al., 2023; Månsson et al., 2021; Nishijima et al., 2022). In the present study, for toddlers diagnosed with ASD who have Bayley cognitive scores < 70 at time of diagnosis, just over half (55.9%) had an IQ score < 70 at early school age (mean age = 74.3 months), and about one-third (38.3%) had an IQ score in the average range or above (>85) at early school age. In comparison, the majority of children with initial Bayley cognitive scores >85 had IQ scores > 85 at early school age. These findings suggest that children with initial lower Bayley cognitive standard scores may have greater variability in future IQ compared to those with initial cognitive scores > 85. Nevertheless, Bayley-III cognitive standard scores in toddlerhood, including both higher and lower levels of function, may provide clinicians with a glimpse into a child with ASD’s possible functioning at early school age, yet they are not predictably associated with the trajectory of cognitive development. Our findings highlight the importance of ongoing monitoring of cognitive development, particularly for children who have low cognitive scores at their initial ASD assessment.

Like other studies on ASD persistence, the present study found that children with non-persistent ASD had significantly higher cognitive scores at the time of initial clinical ASD evaluation compared to children with persistent ASD (Barbaro & Dissanayake, 2017; Chawarska et al., 2009; Elias & Lord, 2022; Hinnebusch et al., 2017; Lord et al., 2006; Moulton et al., 2016; Turner & Stone, 2007; Zachor & Ben-Itzchak, 2020). In addition, our study observed that ASD persistence moderates the change in cognitive trajectories from toddlerhood to early school age. These results are in alignment with the findings of M. L. E. Clark et al. (2017), who also studied changes in cognitive and behavioral profiles of young children with ASD from toddlerhood to school age. As our findings show that core ASD symptoms may change over time and impact cognitive development to different degrees, they emphasize the need for careful ongoing monitoring of overall development to provide targeted treatment recommendations.

This study provided data on the types and intensity of interventions received from the time of the initial clinical ASD diagnosis at 12–36 months until the time of research assessment at approximately 6 years of age. As has been previously reported, there was an overall high rate of intervention received by children in our study, and most children received some ASD-specific interventions (Berg et al., 2024; Harstad et al., 2023). A significantly higher percentage of children with persistent ASD were receiving ABA/ASD-specific interventions from 24 to 66 months after clinical ASD diagnosis, compared to those with non-persistent ASD, who gradually received fewer interventions over time. This likely reflects the decisions to decrease intensity of interventions for children with an initial early ASD diagnosis who make significant developmental progress over time. This pattern of findings is consistent with what was seen by Orinstein et al. (2014) for early interventions received by a sample of 78 individuals diagnosed with ASD before the age of 5 and reassessed at 8–21 years.

The present study demonstrated no relation between receipt of ABA/ASD-specific interventions and change in cognitive trajectories for children with non-persistent ASD and an inverse relationship between rate of change in cognitive trajectories and hours of ABA/ASD-specific interventions received for children with persistent ASD. While this combination of results is inconsistent with the many publications that suggest that receipt of ABA and the ESDM in early childhood leads to notable gains in cognitive functioning (M. Clark et al., 2023; Gitimoghaddam et al., 2022; Godel et al., 2022; Lovaas, 1987; McEachin et al., 1993; Shi et al., 2021), it raises important questions about intensity of ASD services and cognitive outcomes. Given that this was a natural history study where caregivers and clinicians provided recommendations for interventions based on functioning, and almost all children received some ASD-specific interventions following clinical diagnosis, these findings do not indicate that ASD-specific interventions worsen cognitive outcomes. Our results may reflect a trend where children with more severe ASD symptoms and greater cognitive delay are more likely to be referred for higher-intensity interventions. In addition, there was an overall high rate of receipt of interventions by the children in this study, thus potentially making it difficult to find differences in cognitive changes based on interventions received, given that many children received intensive interventions. It should be noted that while it is generally recommended to receive 20–40 h of ABA (Applied Behavior Analysis (ABA)|Autism Speaks, n.d.; Dawson et al., 2010), the children in this study received an average of 7–14 h of ABA at the various 6-month intervals from 0 to 36 months following initial ASD diagnosis in toddlerhood. This was a well-resourced sample representing real-world intervention receipt. While it is possible that stronger effects may have been observed for this sample had the children received higher intensities of intervention, these findings still reflect the impact of real-world intervention receipt on developmental outcomes. Other recent studies investigating the impact of ASD-specific interventions on IQ have shown mixed findings related to intervention intensity; some have found that higher intensities yield greater improvements in IQ, while others have observed no association between intensity and cognitive development (Sandbank et al., 2024; Shi et al., 2021).

When interpreting the findings of this study, there are some limitations to consider. Most children had well-educated, affluent mothers and were White. However, recent publications observed no relationship between socioeconomic factors (including age, gender, household income, primary insurance, and maternal education level) and ASD persistence in this sample (Berg et al., 2024; Harstad et al., 2023). Additionally, this sample of children had a very high level of receipt of ABA/ASD-specific interventions, which is different from what might have been found had the study been conducted in other regions of the United States and is higher than what has been reported in most other studies exploring rates of intervention (i.e., Orinstein et al., 2014).

In conclusion, cognitive functioning in toddlerhood can be predictive of later IQ at school age for young children diagnosed with ASD. Young children with ASD who have initial cognitive scores < 70 show more variation in their early school age IQ scores, compared to those with initial cognitive scores > 85. Children with persistent and non-persistent ASD demonstrate different patterns of cognitive trajectories over time. In this cohort of children with overall high rates of ASD-specific interventions received, the interventions were not associated with changes in cognitive functioning.

## Figures and Tables

**Table 1:** Demographic information for total sample.

Demographic and Sociodemographic Data	Total Sample N (%) (N = 212)
Age at ASD Clinical Diagnosis (months)	
Mean (SD)	24.58 (3.8)
Age at Research Assessment (months)	
Mean (SD)	74.32 (7.1)
Sex	
Female	36 (16.9%)
Male	176 (83.0%)
Race ^a^	
American Indian/Alaskan	1 (0.5%)
Asian	14 (6.6%)
Black/African American	19 (8.9%)
Hawaiian/Pacific Islander	0 (0.0%)
White	170 (80.2%)
Other	18 (8.5%)
Missing/Prefer not to answer	3 (1.4%)
Ethnicity	
Hispanic/Latino	30 (14.2%)
Not Hispanic/Latino	170 (80.2%)
Prefer not to answer	1 (0.5%)
Missing	11 (5.2%)
Insurance Type	
Private	149 (70.3%)
Public	48 (22.6%)
Tri-Care	11 (5.2%)
Self-pay	0 (0.0%)
Other or Unknown ^b^	4 (1.9%)
Maternal Education Level	
≤High school graduate/GED	18 (8.5%)
Trade or vocational school	3 (1.4%)
Associate’s degree	18 (8.5%)
Some college	29 (13.7%)
Bachelor’s degree	67 (31.6%)
Graduate/Professional degree	77 (36.3%)
Prefer not to answer/missing	0 (0.0%)
Annual Household Income	
USD ≤65,999	31 (14.62%)
USD 66,000–100,999	40 (18.87%)
USD 101,000–130,999	32 (15.09%)
USD 131,000–160,999	28 (13.21%)
USD >161,000	59 (27.83%)
Prefer not to answer	21 (9.91%)
Missing	1 (0.5%)

ASD = Autism spectrum disorder. ^a^ N = 12 reported ≥1 race. These categories were provided to the parent who then marked which of the categories best described their child. ^b^ Unknown and missing grouped together on our survey.

**Table 2:** Time 2 (school age) IQ scores as a function of baseline Time 1 (toddler) cognitive scores for total sample *.

Time 1 (Toddler) Cognitive Score	Time 2 (School Age) IQ Score ^a^			
	<70	70– < 85	86– = 100	>100
<70	19	2	9	4
70– < 85	18	9	19	22
86– = 100	9	8	26	58
>100	0	0	1	8

* Note: Given small cell sample sizes, no test statistics were calculated. ^a^ N = 196 completed Differential Ability Scales at Time 2; for the N = 16 who completed Bayley at Time 2, a developmental quotient was calculated as a proxy for Time 2 IQ score. N = 212.

**Table 3:** Percentage of children receiving ABA following clinical ASD diagnosis at 12–36 months of age, separately for persistent versus non-persistent ASD.

Time (in Months) After Initial Clinical ASD Diagnosis	Persistent Total (N = 133) ^a^	Non-Persistent Total (N = 79) ^a^	Chi-Square *p* Value	Effect Size Cramer’s V
0–6	42.9%	39.2%	0.603	0.036
6–12	74.4%	74.7%	0.968	0.003
12–18	80.5%	77.2%	0.574	0.039
18–24	75.2%	64.6%	0.098	0.114
24–30	73.7%	59.5%	0.032 *	0.148
30–36	72.9%	51.9%	0.002 **	0.213
36–42	70.7%	50.6%	0.003 **	0.201
42–48	70.7%	50.6%	0.003 **	0.201
48–54	59.2%	35.5%	0.001 **	0.226
54–60	42.2%	20.8%	0.007 **	0.182
60–66	31.7%	3.1%	0.002 **	0.217

* *p* < 0.05. ** *p* < 0.01. ^a^ From 0 to 48 months, all N = 133 children with persistent ASD and N = 79 children with non-persistent ASD contributed data. Subsequently, there were decreased sample sizes as follows: for 48–54 months, N = 130 persistent and N = 76 non-persistent; for 54–60 months, N = 102 persistent and N = 53 non-persistent; for 60-66 months, N = 60 persistent and N = 32 non-persistent.

**Table 4:** Mixed-effects model effects of hours of ABA on cognitive change scores for children with non-persistent and persistent ASD.

Between-Level Means		
Estimated Parameters ^a^	Non-Persistent ASD	Persistent ASD
0–6 Months Post Clinical ASD Diagnosis		
Mean of ABA Hours	7.127 (1.236) ***	7.348 (0.937) ***
Mean Slope for Children not Receiving ABA (Reference)	18.335 (2.265) ***	3.220 (2.543)
Change in Slope due to ABA	0.036 (0.121)	−0.227 (0.210)
6–12 Months Post Clinical ASD Diagnosis		
Mean of ABA Hours	13.443 (1.395) ***	12.821 (0.963) ***
Mean Slope for Children not Receiving ABA (Reference)	20.315 (2.674) ***	1.920 (3.030)
Change in Slope due to ABA	−0.128 (0.118)	−0.029 (0.190)
12–18 Months Post Clinical ASD Diagnosis		
Mean of ABA Hours	13.620 (1.393) ***	13.964 (0.988) ***
Mean Slope for Children not Receiving ABA (Reference)	17.626 (2.996) ***	6.986 (3.112) *
Change in Slope due to ABA	0.071 (0.141)	−0.389 (0.176) *
18–24 Months Post Clinical ASD Diagnosis		
Mean of ABA Hours	10.101 (1.312) ***	12.585 (1.021) ***
Mean Slope for Children not Receiving ABA (Reference)	17.488 (2.530) ***	7.536 (3.043) *
Change in Slope due to ABA	0.110 (0.145)	−0.476 (0.185) **
24–30 Months Post Clinical ASD Diagnosis		
Mean of ABA Hours	9.513 (1.332) ***	11.789 (0.997) ***
Mean Slope for Children not Receiving ABA (Reference)	17.943 (2.476) ***	9.505 (2.825) **
Change in Slope due to ABA	0.069 (0.144)	−0.675 (0.187) ***
30–36 Months Post Clinical ASD Diagnosis		
Mean of ABA Hours	8.348 (1.291) ***	11.530 (1.002) ***
Mean Slope for Children not Receiving ABA (Reference)	18.462 (2.370) ***	9.252 (2.834) **
Change in Slope due to ABA	0.016 (0.148)	−0.668 (0.174) ***

Note: Values are estimates with standard errors in parentheses. Significant values were corrected using the Benjamini–Hochberg corrective procedure and a false discovery rate of 5%. * *p* < 0.05. ** *p* < 0.01. *** *p* < 0.001. ^a^ At all 6-month time intervals from 0 to 36 months post clinical ASD diagnosis, the intercept of cognitive scores for children with non-persistent ASD (86.0, SE = 1.3) was significantly higher than for children with persistent ASD (79.1, SE = 1.3), z = 3.80, *p* < 0.001.

**Table 5:** Mixed-effects model effects of hours of any ASD-specific interventions on cognitive change scores for children with non-persistent and persistent ASD.

Between-Level Means		
Estimated Parameters ^a^	Non-Persistent ASD	Persistent ASD
0–6 Months Post Clinical ASD Diagnosis		
Mean of ASD-Specific Interventions in Hours	7.148 (1.236) ***	7.844 (0.960) ***
Mean Slope for Children not Receiving ASD-Specific Interventions (Reference)	18.332 (2.269) ***	3.000 (2.597)
Change in Slope due to ASD-Specific Interventions	0.037 (0.121)	−0.185 (0.205)
6–12 Months Post Clinical ASD Diagnosis		
Mean of ASD-Specific Interventions in Hours	13.489 (1.393) ***	13.397 (0.967) ***
Mean Slope for Children not Receiving ASD-Specific Interventions (Reference)	20.358 (2.682) ***	2.133 (3.138)
Change in Slope due to ASD-Specific Interventions	−0.131 (0.118)	−0.043 (0.188)
12–18 Months Post Clinical ASD Diagnosis		
Mean of ASD-Specific Interventions in Hours	13.658 (1.390) ***	14.205 (0.986) ***
Mean Slope for Children not Receiving ASD-Specific Interventions (Reference)	17.646 (3.009) ***	7.326 (3.160) *
Change in Slope due to ASD-Specific Interventions	0.069 (0.142)	−0.407 (0.176) *
18–24 Months Post Clinical ASD Diagnosis		
Mean of ASD-Specific Interventions in Hours	10.158 (1.309) ***	12.825 (1.022) ***
Mean Slope for Children not Receiving ASD-Specific Interventions (Reference)	17.500 (2.544) ***	7.827 (3.082) *
Change in Slope due to ASD-Specific Interventions	0.108 (0.146)	−0.489 (0.184) **
24–30 Months Post Clinical ASD Diagnosis		
Mean of ASD-Specific Interventions in Hours	9.570 (1.329) ***	11.797 (0.998) ***
Mean Slope for Children not Receiving ASD-Specific Interventions (Reference)	17.958 (2.488) ***	9.549 (2.822) **
Change in Slope due to ASD-Specific Interventions	0.067 (0.145)	−0.678 (0.187) ***
30–36 Months Post Clinical ASD Diagnosis		
Mean of ASD-Specific Interventions in Hours	8.386 (1.290) ***	11.538 (1.002) ***
Mean Slope for Children not Receiving ASD-Specific Interventions (Reference)	18.479 (2.376) ***	9.295 (2.830) **
Change in Slope due to ASD-Specific Interventions	0.014 (0.148)	−0.671 (0.175) ***

Note: Values are estimates with standard errors in parentheses. Significant values were corrected using the Benjamini–Hochberg corrective procedure and a false discovery rate of 5%. * *p* < 0.05. ** *p* < 0.01. *** *p* < 0.001. ^a^ At all 6-month time intervals from 0 to 36 months post clinical ASD diagnosis, the intercept of cognitive scores for children with non-persistent ASD (86.0, SE = 1.3) was significantly higher than for children with persistent ASD (79.1, SE = 1.3), z = 3.80, *p* < 0.001.

## Data Availability

This dataset is not publicly available due to privacy restrictions. The raw data supporting the conclusions of this article will be made available by the authors on request.

## Supplementary Materials

The following supporting information can be downloaded at https://www.mdpi.com/article/10.3390/bs15111445/s1, Table S1: Percentage of children receiving ASD-specific interventions following clinical ASD diagnosis at 12–36 months of age, separately for persistent versus non-persistent ASD.

## Abbreviations

The following abbreviations are used in this manuscript:

ASD	Autism spectrum disorder
Bayley-III	Bayley Scales of Infant and Toddler Development, Third Edition
IQ	Intellectual quotient
ABA	Applied Behavioral Analysis
ESDM	Early Start Denver Model
DSM-5	Diagnostic and Statistical Manual of Mental Disorders, Fifth Edition
IRB	Institutional review board
DBP	Developmental–behavioral pediatrician
ADOS-2	Autism Diagnostic Observation Schedule, Second Edition
ADI-R	Autism Diagnostic Interview-Research
DAS-II	Differential Abilities Scales, Second Edition
SCERTS	Social Communication/Emotional Regulation/Transactional Support
RDI	Relationship Developmental Intervention
