# Supplementary material for: Cognitive Development in Children with Autism Spectrum Disorder and the Moderating Role of Intervention and ASD Persistence
Source: Behav Sci (Basel). 2025 Oct 23;15(11):1445. doi: 10.3390/bs15111445 (PMC12649235; doi:10.3390/bs15111445)
Supplement: Supplementary file 1 [file behavsci-15-01445-s001.zip › behavsci-3874388-supplementary.pdf]

**Supplemental Table S1.** Percentage of children receiving ASD-specific interventions following clinical ASD diagnosis at 12-36 months of age, separately for persistent versus non-persistent ASD.

| Time (in Months) After Initial Clinical ASD Diagnosis | Persistent Total (N=133)[a] | Non-persistent Total (N=79)[a] | Chi-Square *p* Value | Effect Size Cramer's V |
|---|---|---|---|---|
| 0-6 | 45.9% | 40.5% | .447 | 0.052 |
| 6-12 | 77.4% | 75.9% | .803 | 0.017 |
| 12-18 | 81.2% | 78.5% | .631 | 0.033 |
| 18-24 | 75.9% | 65.8% | .112 | 0.109 |
| 24-30 | 73.7% | 60.8% | .049* | 0.135 |
| 30-36 | 72.9% | 53.2% | .003** | 0.201 |
| 36-42 | 70.7% | 50.6% | .003** | 0.201 |
| 42-48 | 70.7% | 50.6% | .003** | 0.201 |
| 48-54 | 59.2% | 35.5% | .001** | 0.226 |
| 54-60 | 42.2% | 20.8% | .007** | 0.182 |
| 60-66 | 31.7% | 3.1% | .002** | 0.217 |

* $p < 0.05$. ** $p < 0.01$. [a] From 0 to 48 months, all N = 133 children with persistent ASD and N = 79 children with non-persistent ASD contributed data. Subsequently, there were decreased sample sizes as follows: for 48–54 months, N = 130 persistent and N = 76 non-persistent; for 54–60 months, N = 102 persistent and N = 53 non-persistent; for 60-66 months, N = 60 persistent and N = 32 non-persistent.
